# Multi-Focal Symptom-Based Neuromodulation for Memory and Depression in Dementia

**DOI:** 10.1093/geroni/igaf122.2272

**Published:** 2025-12-31

**Authors:** Jasmine Morgan, Alisha Syed, Wanting Yu, Judy Zheng, Alexander Opitz, Brad Manor, Alvaro Pascual-Leone, Davide Cappon

**Affiliations:** Hebrew SeniorLife - Marcus Institute for Aging Research, Boston, Massachusetts, United States; Hebrew SeniorLife, Boston, Massachusetts, United States; Hebrew SeniorLife, Boston, Massachusetts, United States; Marcus Institute for Aging Research, Roslindale, Massachusetts, United States; University of Minnesota, Minneapolis, Minnesota, United States; Hebrew SeniorLife/Harvard Medical School, Boston, Massachusetts, United States; Hebrew SeniorLife & Harvard Medical School, Boston, Massachusetts, United States; Hebrew SeniorLife & Harvard Medical School, Boston, Massachusetts, United States

## Abstract

Major depressive disorder in the context of dementia (MDD + D) is highly prevalent, on the rise, and a leading cause of impairment in older adults. It accelerates functional decline, worsens health outcomes, hastens long-term care admission, increases caregiver burden, and raises mortality risk. MDD+D involves brain network dysfunction affecting mood and memory. Transcranial direct current stimulation (tDCS) improves mood in MDD, while transcranial alternating current stimulation (tACS) enhances memory in dementia. Combining these neuromodulation techniques offers a promising dual-targeted intervention. This study evaluates the safety and feasibility of a home-based, caregiver-administered tACS + tDCS intervention. Ten participants (8 female, average age 73 ± 7.13) underwent a 4-week intervention consisting of 20-minute sessions, five days per week, targeting the left prefrontal cortex (tDCS) and left angular gyrus (tACS). Caregivers, trained by study staff, administered the stimulation with initial tele-supervision before continuing independently with remote monitoring. Participants completed questionnaires to assess safety and feasibility. Following the intervention, participants assessed their experience. All ten participants completed the study, with eight adhering to 100% of sessions, one completing 95%, and one 90%. Across 197 total sessions, the most reported side effect was scalp sensations (tingling, itching), mild in 55%, moderate in 21%, and severe in 1%. 90% of participants expressed willingness to repeat the program and continue for a longer duration. A home-based tACS + tDCS program is feasible, safe, and well-tolerated, with high adherence and satisfaction, supporting further investigation into at-home tACS + tDCS treatment for MDD+D.

